# First report of *Dicopus longipes* (Subba Rao) (Hymenoptera: Chalcidoidea) from India with new distribution data on some species

**DOI:** 10.3897/BDJ.3.e4692

**Published:** 2015-03-10

**Authors:** A. Rameshkumar, J. Poorani, M. Anjana

**Affiliations:** ‡ ICAR-National Bureau of Agricultural Insect Resources, Bangalore 560024, India; § Western Ghats Regional Centre, Zoological Survey of India, Calicut 673006, India

**Keywords:** Mymaridae, distribution, India

## Abstract

*Dicopus longipes* (Subba Rao) (Hymenoptera: Chalcidoidea: Mymaridae) is recorded from India for the first time. New additional distribution records of Mymaridae from the southern Indian states of Tamil Nadu and Kerala are documented.

## Introduction

Fairyflies (Hymenoptera: Chalcidoidea: Mymaridae) are internal egg parasitoids of insects except two species that parasitize the larvae of eulophids (Huber et al. 2006). From India, 31 genera and 134 species of Mymaridae have been reported so far (Manickavasagam and Rameshkumar 2013, Ramesh Kumar et al. 2013). The mymarid fauna of India is not well documented as several states and biodiversity rich areas of India have not been surveyed so far for mymarids. Because mymarids are small to tiny, only Malaise traps and yellow pan traps yield good field collections. In this paper, we document new records of mymarids for the southern Indian states of Kerala and Tamil Nadu. One species, *Dicopus longipes* (Subba Rao), is reported as new to India.

## Materials and methods

Parasitoids were collected from Kasaragod, Kozhikode, Palakkad, and Idukki districts of Kerala and Coimbatore and Salem districts of Tamil Nadu using sweep netting, yellow pan traps and Malaise traps in different ecosystems (Noyes 1982). Collected specimens were processed using hexamethyldisilazane (HMDS) (Brown 1993) and card/slide mounted for identification. Voucher specimens are deposited in the reference collections of the National Bureau of Agricultural Insect Resources (ICAR-NBAIR), Bangalore, India and the Zoological Survey of India (ZSI), Western Ghat Regional Centre, Calicut, India.

## Taxon treatments

### Acmopolynema indochinense

Soyka

#### Materials

- **Type status:** Other material. **Occurrence:** recordedBy: Kumar; individualCount: 1; sex: female; lifeStage: Adult; **Location:** continent: Asia; country: India; countryCode: IND; stateProvince: Kerala; municipality: Palakkad; locality: Chittur; **Identification:** identifiedBy: A Rameshkumar; **Event:** samplingProtocol: Yellow pan trap; eventDate: 2011-02-04; **Record Level:** institutionID: ICAR-National Bureau of Agricultural Insect Resources; institutionCode: ICAR-NBAIR

#### Distribution

**Figure 1.**
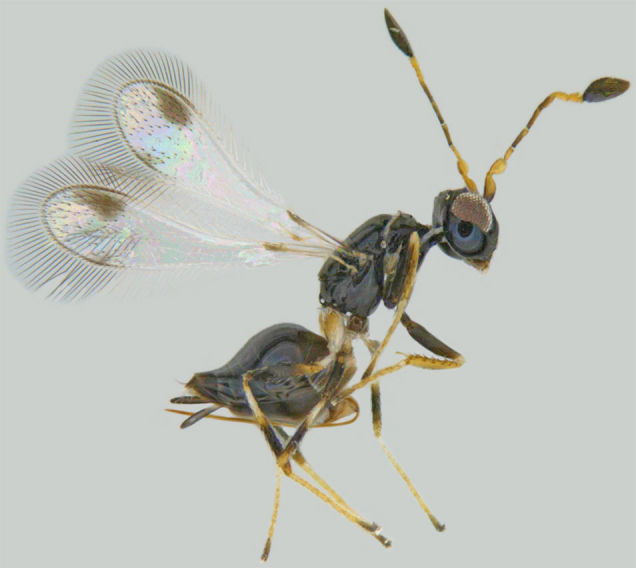
*Acmopolynema indochinense*, lateral view.

*Acmopolynema indochinense* (Fig. 1​), hitherto known from Karnataka and Uttar Pradesh (Triapitsyn and Berezovskiy 2007), is a new record for Kerala.

### Acmopolynema malabaricum

Subba Rao

#### Materials

- **Type status:** Other material. **Occurrence:** recordedBy: A Rameshkumar; individualCount: 4; sex: females; lifeStage: Adult; **Location:** continent: Asia; country: India; countryCode: IND; stateProvince: Tamil Nadu; municipality: Salem; locality: Yercaud; **Identification:** identifiedBy: A Rameshkumar; **Event:** samplingProtocol: Yellow pan trap; eventDate: 2014-08-06; **Record Level:** institutionID: ICAR-National Bureae of Agricultural Insect Resources; institutionCode: ICAR-NBAIR

#### Distribution

**Figure 2.**
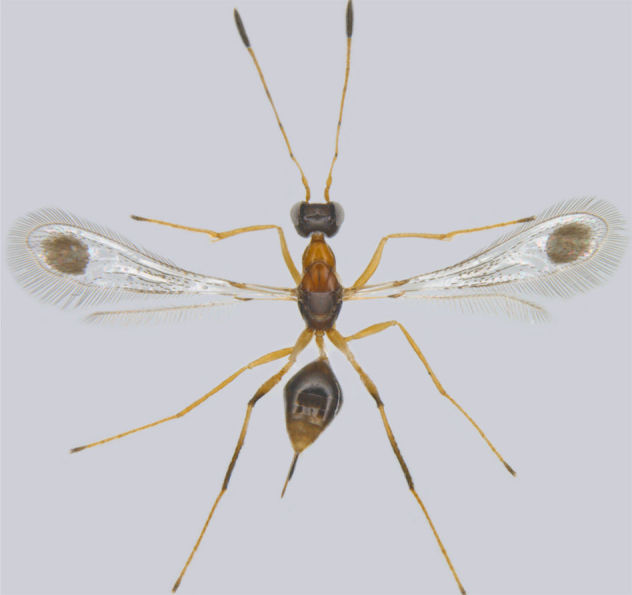
*Acmopolynema malabaricum*, lateral view.

*Acmopolynema malabaricum* (Fig. 2​) was known only from Kerala (Hayat and Anis 1999) and is new to Tamil Nadu.

### Acmopolynema problema

Triapitsyn & Berezovskiy

#### Materials

- **Type status:** Other material. **Occurrence:** recordedBy: Nikhil; individualCount: 1; sex: female; lifeStage: Adult; **Location:** continent: Asia; country: India; countryCode: IND; stateProvince: Kerala; municipality: Palakkad; locality: near Silent Valley; **Identification:** identifiedBy: A Rameshkumar; **Event:** samplingProtocol: Sweep net; eventDate: 2013-01-16; habitat: Weedy field; **Record Level:** institutionID: ICAR-National Bureau of Agricultural Insect Resources; institutionCode: ICAR-NBAIR

#### Distribution

**Figure 3.**
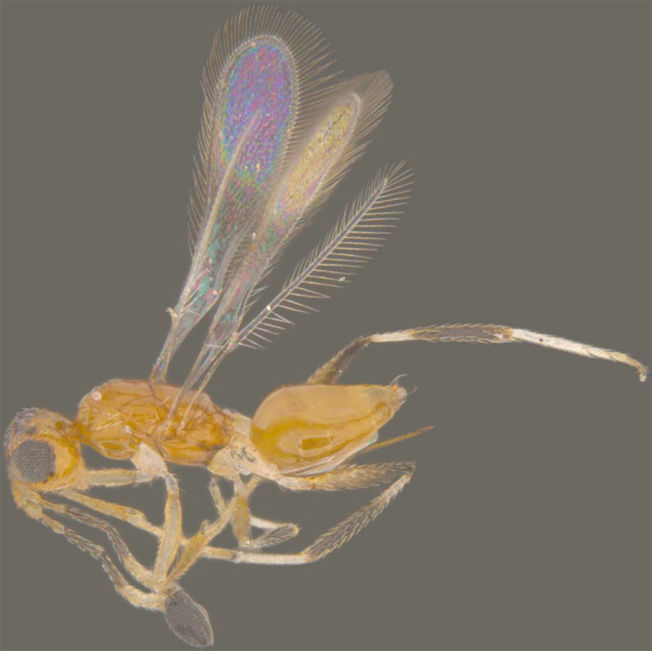
*Acmopolynema problema*, lateral view.

*Acmopolynema problema* (Fig. 3​​) has been recorded from only Karnataka (Triapitsyn and Berezovskiy 2007) and is recorded here from Kerala.

### Alaptus sp.

#### Materials

- **Type status:** Other material. **Occurrence:** recordedBy: A Rameshkumar; individualCount: 5; sex: female; lifeStage: Adult; **Location:** continent: Asia; country: India; countryCode: IND; stateProvince: Kerala; municipality: Palakkad; locality: Pattambi; verbatimLocality: RARS campus; **Identification:** identifiedBy: A Rameshkumar; **Event:** samplingProtocol: Yellow pan trap; eventTime: 2014-01-25; habitat: Paddy field; **Record Level:** institutionID: ICAR-National Bureau of Agricultural Insect Resources; institutionCode: ICAR-NBAIR

#### Distribution

**Figure 4.**
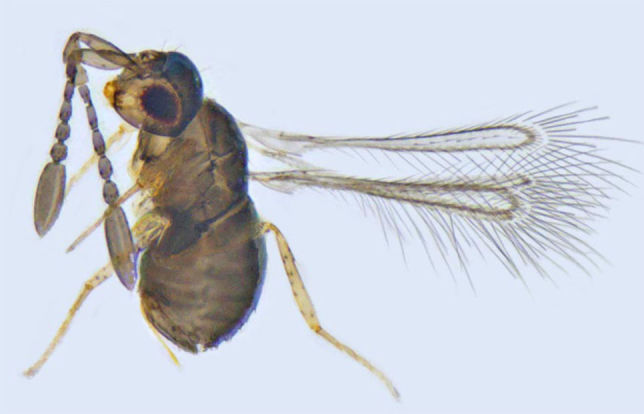
*Alaptus* sp., lateral view.

*Alaptus* (Fig. 4​) includes some of the smallest mymarids. From India, it was recorded from Delhi, West Bengal, Tamil Nadu (Subba Rao and Hayat 1983), Karnataka (Manickavasagam et al. 2011), and Pudhucherry (Rameshkumar et al. 2011). We record it here from Kerala.

### Camptoptera matcheta

Subba Rao

#### Materials

- **Type status:** Other material. **Occurrence:** recordedBy: A Rameshkumar; individualCount: 3; sex: female; lifeStage: Adult; **Location:** continent: Asia; country: India; countryCode: IND; stateProvince: Tamil Nadu; municipality: Salem; locality: Yercaud; **Identification:** identifiedBy: A Rameshkumar; **Event:** samplingProtocol: Yellow pan trap; eventDate: 2014-08-06; habitat: Weedy filed; **Record Level:** institutionID: ICAR-National Bureau of Agricultural Insect Resources; institutionCode: ICAR-NBAIR

#### Distribution

**Figure 5.**
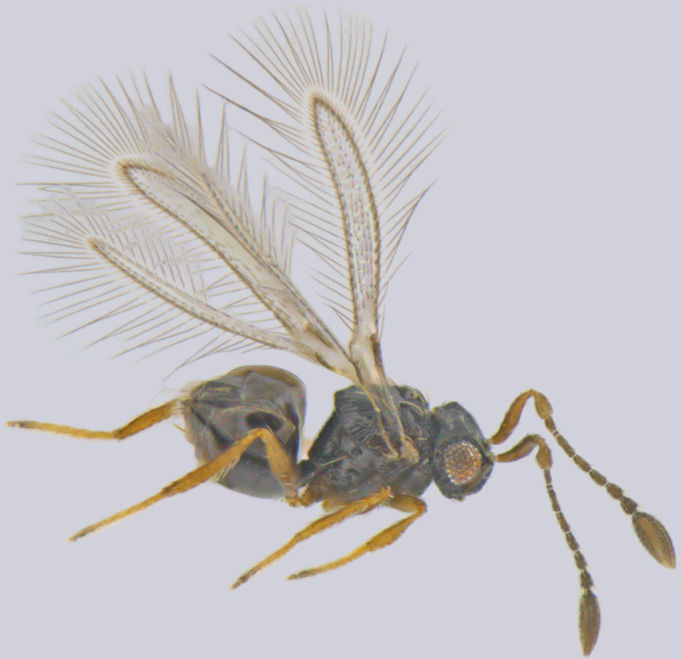
*Camptoptera matcheta*, lateral view.

*Camptoptera matcheta* (Fig. 5​) was known only from Karnataka (Subba Rao 1989) and is new to Tamil Nadu.

### Dicopus longipes

(Subba Rao)

#### Materials

- **Type status:** Other material. **Occurrence:** recordedBy: A Rameshkumar; individualCount: 1; sex: Female; lifeStage: Adult; **Location:** continent: Asia; country: India; countryCode: IND; stateProvince: Kerala; municipality: Palakkad; locality: RARS campus; **Identification:** identificationID: A Rameshkumar; **Event:** samplingProtocol: Yellow pan trap; eventDate: 2014-01-25; habitat: Paddy field; **Record Level:** institutionID: ICAR-National Bureau of Agricultural Insect Resources, Bangalore; institutionCode: ICAR-NBAIR

#### Diagnosis

**Figure 6.**
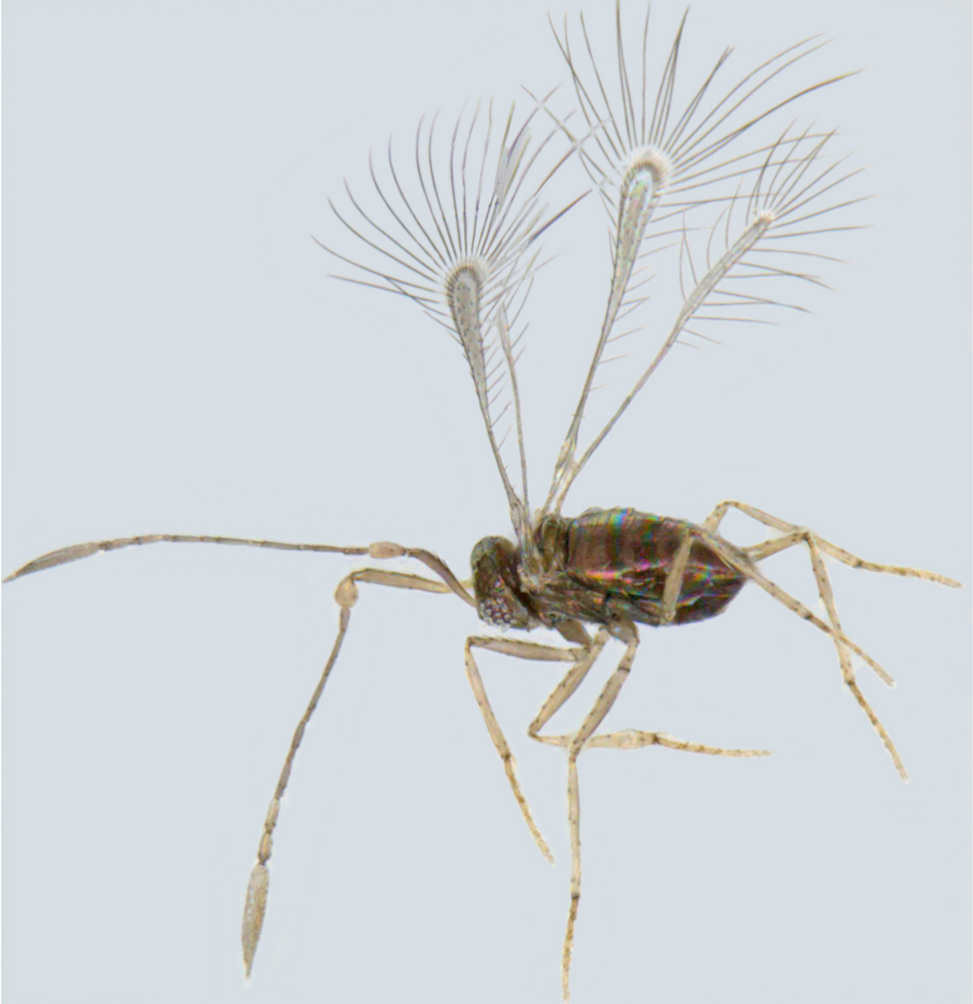
*Dicopus longipes*, lateral view.

*Dicopus longipes* (Fig. 6​​) differs from *D. noyesi* Manickavasagam by the following features: antennal scape uniformly slender (distinctly bulging subapically in *D. noyesi*), F1 almost as long as F2 (distinctly shorter in *D. noyesi*), F6 and F7 slightly bottle shaped, the apex of each segment shorter and wider (F5 to F7 bottle-neck shaped, the apex of each segment distinctly long and narrow in *​D. noyesi*).

#### Distribution

**​** Malaysia (Subba Rao 1984); India (Kerala). Pricop and Andriescu (2011) have mentioned that *D. longipes* is known from India, but it appears to be incorrect. After Subba Rao (1984) described it from Malaysia-Sabah under *Kubja*, the only subsequent reference to this species was by Huber (2009) when he synonymised *Kubja* with *Dicopus*.

### Dicopus noyesi

Manickavasagam

#### Materials

- **Type status:** Other material. **Occurrence:** recordedBy: A Rameshkumar; individualCount: 2; sex: female; lifeStage: Adult; **Location:** continent: Asia; country: India; countryCode: IND; stateProvince: Kerala; municipality: Calicut; locality: red hills; **Identification:** identifiedBy: A Rameshkumar; **Event:** samplingProtocol: Yellow pan trap; eventDate: 2014-04-25; habitat: Weedy field; **Record Level:** institutionID: ICAR-National Bureau of Agricultural Insect Resources; institutionCode: ICAR-NBAIR

#### Distribution

**Figure 7.**
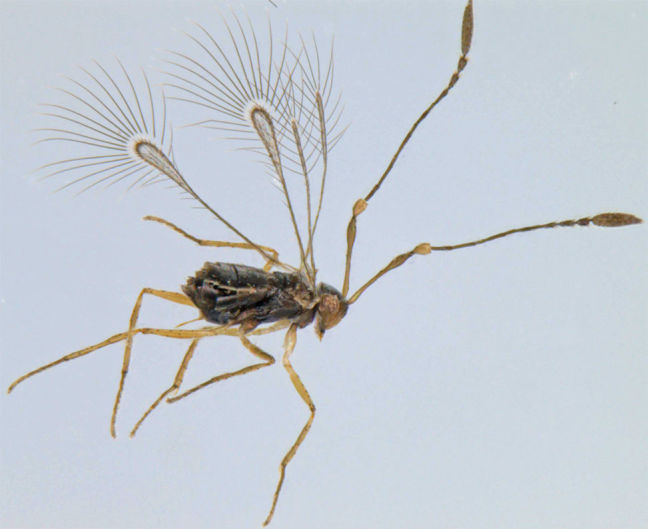
*Dicopus noyesi*, lateral view.

*Dicopus noyesi* (Fig. 7​) was recorded from Andhra Pradesh and Tamil Nadu (Manickavasagam and Rameshkumar 2011) and we record it here from Kerala.

### Eofoersteria sp.

#### Materials

- **Type status:** Other material. **Occurrence:** recordedBy: A Rameshkumar; individualCount: 11; sex: female; lifeStage: Adult; **Location:** continent: Asia; country: India; countryCode: IND; stateProvince: Kerala; municipality: Palakkad; locality: Pattambi; verbatimLocality: RARS campus; **Identification:** identifiedBy: A Rameshkumar; **Event:** samplingProtocol: Yellow pan trap; eventDate: 2014-01-25; habitat: Paddy field; **Record Level:** institutionID: ICAR-National Bureau of Agricultural Insect Resources; institutionCode: ICAR-NBAIR
- **Type status:** Other material. **Occurrence:** recordedBy: A Rameshkumar; individualCount: 1; sex: female; lifeStage: Adult; **Location:** continent: Asia; country: India; countryCode: IND; stateProvince: Kerala; municipality: Calicut; locality: Mavoor; verbatimLocality: Medical college campus; **Identification:** identifiedBy: A Rameshkumar; **Event:** samplingProtocol: Yellow pan trap; eventDate: 2012-11-22; habitat: grassy field; **Record Level:** institutionID: ICAR-National Bureau of Agricultural Insect Resources; institutionCode: ICAR-NBAIR

#### Distribution

**Figure 8.**
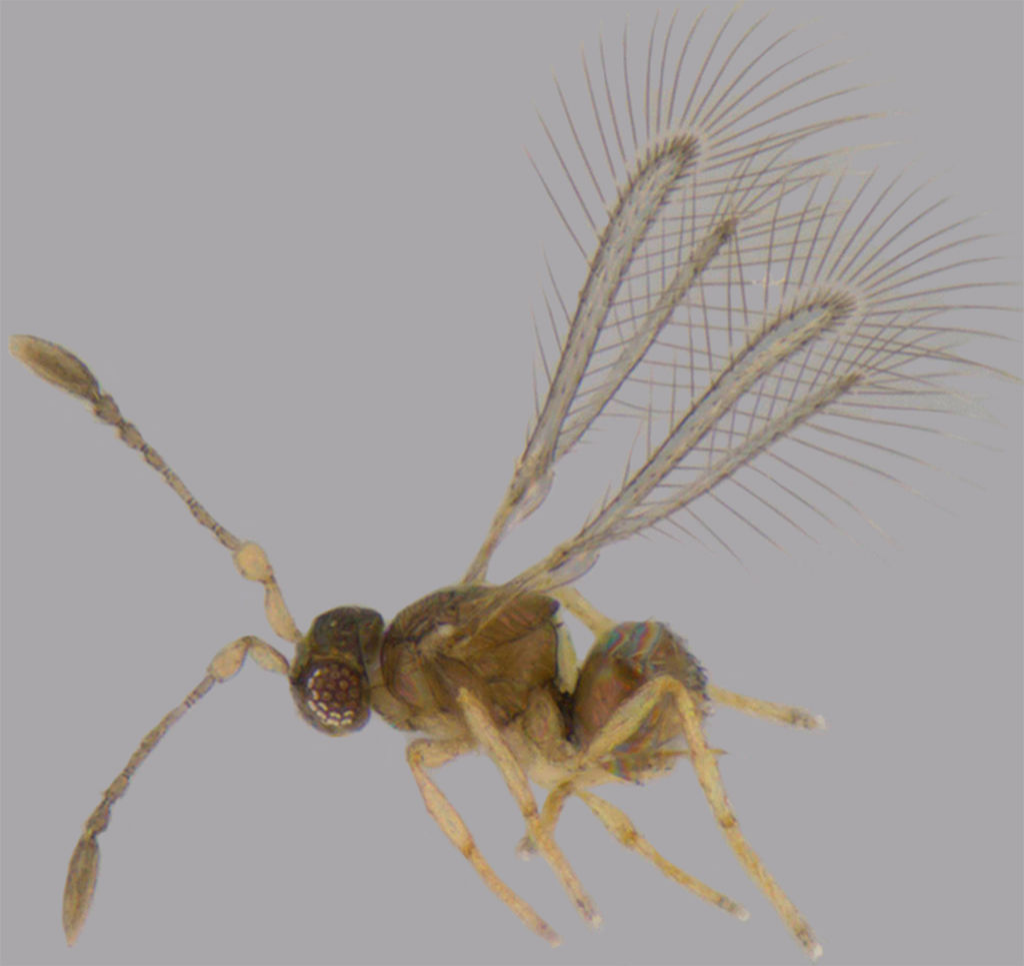
*Eofoersteria* sp., lateral view.

*Eofoersteria* (Fig. 8​) was recorded from Tamil Nadu (Subba Rao and Hayat 1983) and Karnataka (Manickavasagam et al. 2011). We record it here from Kerala.

### Eubroncus sp.

#### Materials

- **Type status:** Other material. **Occurrence:** recordedBy: A Rameshkumar; individualCount: 1, 1; sex: male, female; lifeStage: Adult; **Location:** continent: Asia; country: India; countryCode: IND; stateProvince: Tamil Nadu; municipality: Valparai; locality: Urulikkal; verbatimLocality: Periyar nagar; **Identification:** identifiedBy: A Rameshkumar; **Event:** samplingProtocol: Yellow pan trap; eventTime: 2014-05-04; habitat: Weedy field; **Record Level:** institutionID: ICAR-National Bureau of Agricultural Insect Resources; institutionCode: ICAR-NBAIR

#### Distribution

**Figure 9.**
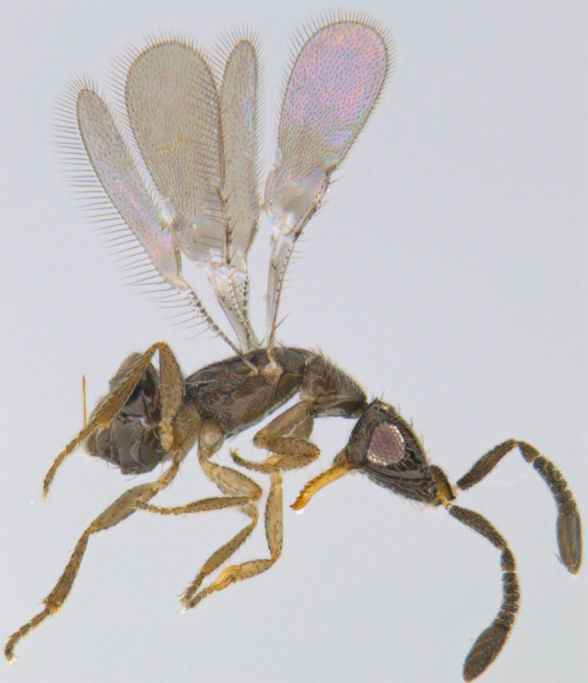
*Eubroncus* sp., lateral view.

*Eubroncus* (Fig. 9​​) is a rarely collected genus previously known from West Bengal (Hayat and Khan 2009) and Karnataka (Manickavasagam et al. 2011). We record it here from Tamil Nadu.

### Gonatocerus monticolus

Zeya

#### Materials

- **Type status:** Other material. **Occurrence:** recordedBy: Raseena; individualCount: 1; sex: female; lifeStage: Adult; **Location:** continent: Asia; country: India; countryCode: IND; stateProvince: Kerala; municipality: Calicut; locality: Vengeri; **Identification:** identifiedBy: A Rameshkumar; **Event:** samplingProtocol: Malaise trap; eventTime: 2014-02-13; habitat: Weedy field; **Record Level:** institutionID: ICAR-National Bureau of Agricultural Insect Resources; institutionCode: ICAR-NBAIR

#### Distribution

**Figure 10.**
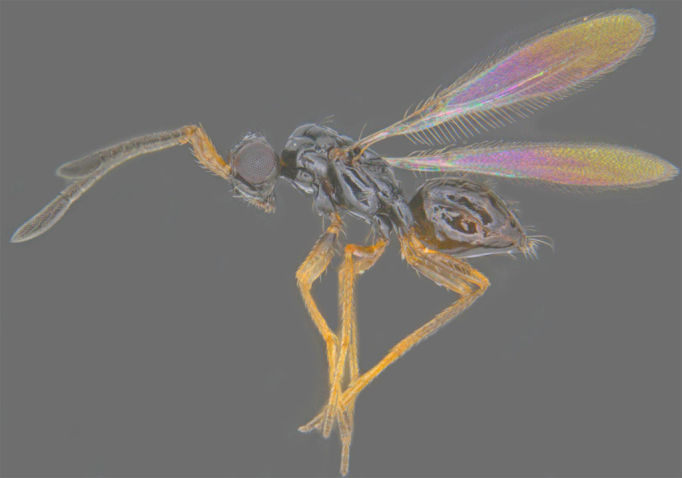
*Gonatocerus monticolus*, lateral view.

*Gonatocerus monticolus* (Fig. 10​​) is known only from its type locality in Uttar Pradesh (Zeya and Hayat 1995). We record it here from Kerala.

### Gonatocerus trialbifuniculatus

Subba Rao

#### Materials

- **Type status:** Other material. **Occurrence:** recordedBy: Raseena; individualCount: 1; sex: female; lifeStage: Adult; **Location:** continent: Asia; country: India; countryCode: IND; stateProvince: Kerala; municipality: Calicut; locality: Vengeri; **Identification:** identifiedBy: A Rameshkumar; **Event:** samplingProtocol: Malaise trap; eventDate: 2014-02-13; habitat: Weedy field; **Record Level:** institutionID: ICAR-National Bureau of Agricultural Insect Resources; institutionCode: ICAR-NBAIR

#### Distribution

**Figure 11.**
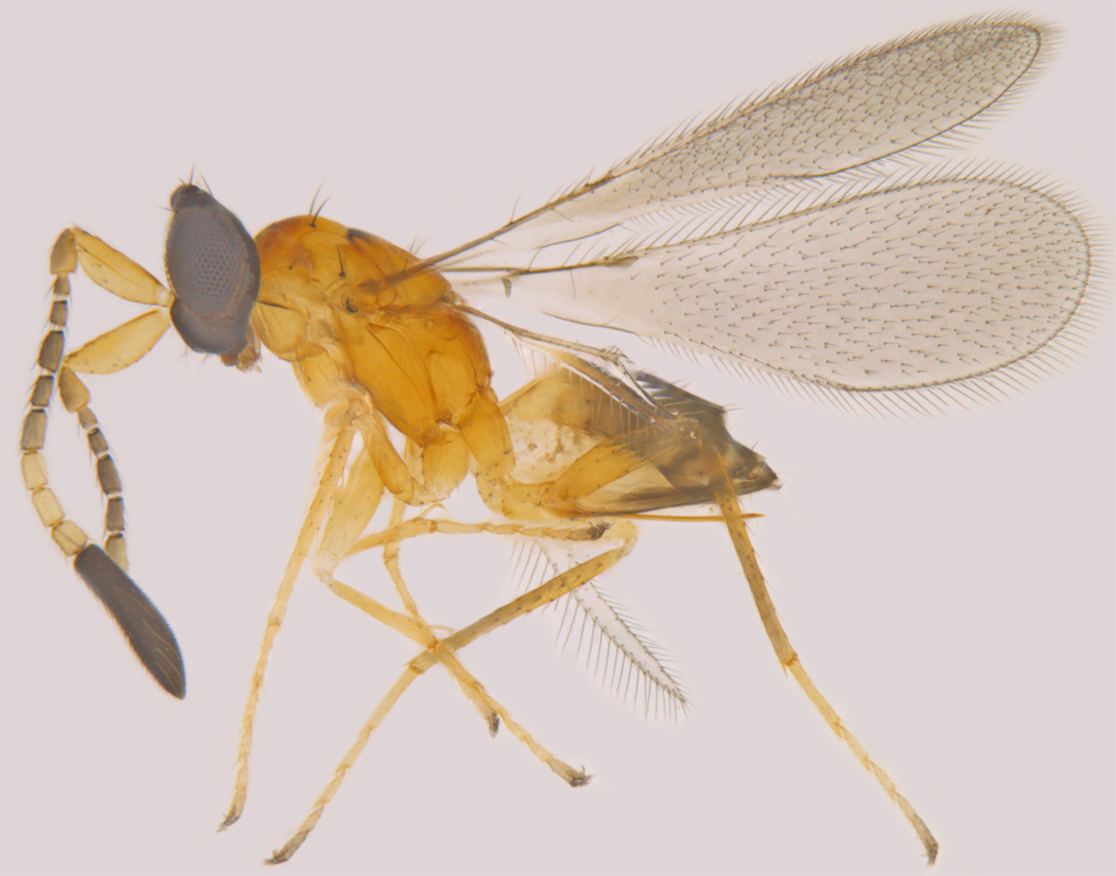
*Gonatocerus trialbifuniculatus*, lateral view.

*Gonatocerus trialbifuniculatus* (Fig. 11​) is known only from Karnataka (Zeya and Hayat 1995). We record it here from Kerala.

### Kikiki huna

Huber

#### Materials

- **Type status:** Other material. **Occurrence:** recordedBy: A Rameshkumar; individualCount: 24; sex: females; lifeStage: Adult; **Location:** continent: Asia; country: India; countryCode: IND; stateProvince: Tamil Nadu; municipality: Salem; locality: Yercaud; **Identification:** identifiedBy: A Rameshkumar; **Event:** samplingProtocol: Yellow pan trap; eventDate: 2014-08-06; habitat: Weedy field; **Record Level:** institutionID: ICAR-National Bureau of Agricultural Insect Resources; institutionCode: ICAR-NBAIR

#### Distribution

Costa Rica, Hawaiian Islands, Trinidad and Tobago (Huber and Noyes 2013); Argentina (Triapitsyn 2013​); India (Tamil Nadu).

#### Taxon discussion

*Kikiki* is unique among Mymaridae in having 4-segmented funicle, 2-segmented clava and 3-segemented tarsi. Only the type species, *K. huna* Huber (Fig. 12), is known so far. In India, *Kikiki* was first recorded by Manickavasagam and Palanivel (2013) from Tamil Nadu, but they did not confirm the species identity. The size of *K. huna* ranges from 150 to 170 µm and it holds the record for being the smallest winged insect known at present (Huber and Noyes 2013).

### Litus sutil

Triapitsyn and Berezovskiy

#### Materials

- **Type status:** Other material. **Occurrence:** recordedBy: A Rameshkumar; individualCount: 1; sex: female; lifeStage: Adult; **Location:** continent: Asia; country: India; countryCode: IND; stateProvince: Tamil Nadu; municipality: Salem; locality: Yercaud; **Identification:** identifiedBy: A Rameshkumar; **Event:** samplingProtocol: Yellow pan trap; eventDate: 2014-08-06; habitat: Weedy field; **Record Level:** institutionID: ICAR-National Bureau of Agricultural Insect Resources; institutionCode: ICAR-NBAIR

#### Distribution

**Figure 13.**
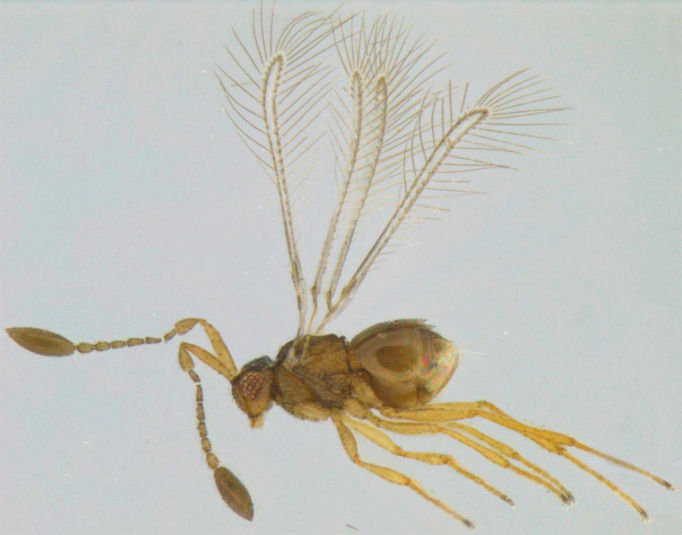
*Litus sutil*, lateral view.

*Litus sutil* (Fig. 13​) was known from Meghalaya (Ramesh Kumar et al. 2013). We record it here from Tamil Nadu.

### Litus triapitsyni

Rehmat and Hayat

#### Materials

- **Type status:** Other material. **Occurrence:** recordedBy: A Rameshkumar; individualCount: 1; sex: female; lifeStage: Adult; **Location:** continent: Asia; country: India; countryCode: IND; municipality: Salem; locality: Yercaud; **Identification:** identifiedBy: A Rameshkumar; **Event:** samplingProtocol: Yellow pan trap; eventTime: 2014-08-06; habitat: Weedy field; **Record Level:** institutionID: ICAR-National Bureau of Agricultural Insect Resources; institutionCode: ICAR-NBAIR

#### Distribution

**Figure 14.**
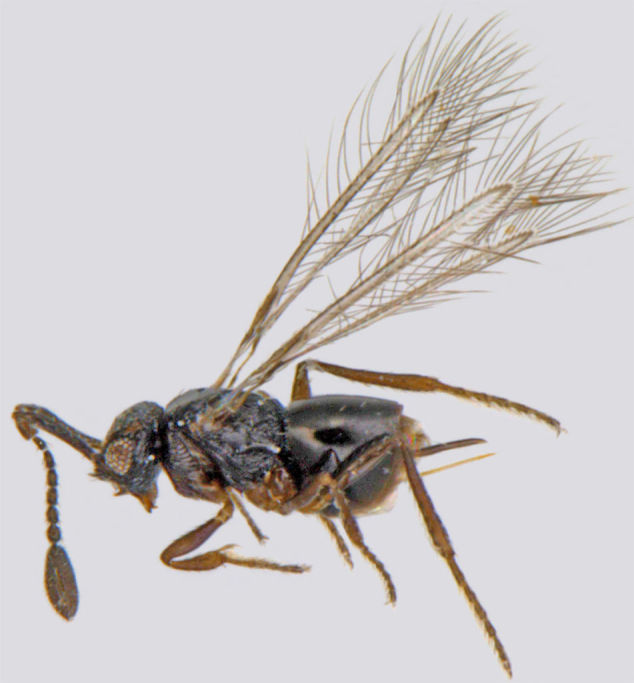
*Litus triapitsyni*, lateral view.

*Litus triapitsyni* Rehmat and Hayat (Fig. 14​) was recorded from Assam (Rehmat et al. 2009). We record it here from Tamil Nadu.

### Ooctonus nigrotestaceus

Subba Rao

#### Materials

- **Type status:** Other material. **Occurrence:** recordedBy: Abhilash; individualCount: 1; sex: female; lifeStage: Adult; **Location:** continent: Asia; country: India; countryCode: IND; stateProvince: Kerala; municipality: Idukki,; locality: Vellimala; **Identification:** identifiedBy: A Rameshkumar; **Event:** samplingProtocol: Sweep net; eventDate: 2013-04-07; habitat: Weedy field; **Record Level:** institutionID: ICAR-National Bureau of Agricultural Insect Resources; institutionCode: ICAR-NBAIR
- **Type status:** Other material. **Occurrence:** recordedBy: Bijoy; individualCount: 1; sex: female; lifeStage: Adult; **Location:** continent: Asia; country: India; countryCode: IND; stateProvince: Kerala; municipality: Idukki; locality: Mannavan shola; **Identification:** identifiedBy: A Rameshkumar; **Event:** samplingProtocol: Malaise trap; eventDate: 2013-04-07; habitat: Weedy field; **Record Level:** institutionID: ICAR-National Bureau of Agricultural Insect Resources; institutionCode: ICAR-NBAIR

#### Distribution

**Figure 15.**
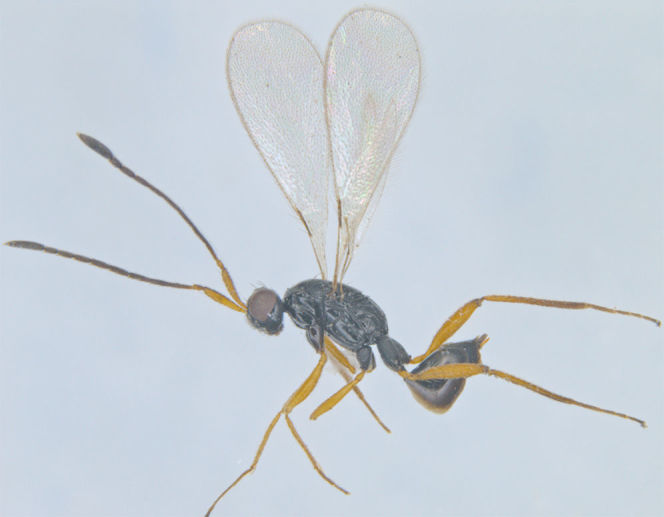
*Ooctonus nigrotestaceus*, lateral view.

*Ooctonus nigrotestaceus* Subba Rao (Fig. 15​) was originally described from Karnataka (Subba Rao 1989). We record it here from Kerala.

### Schizophragma sp.

#### Materials

- **Type status:** Other material. **Occurrence:** recordedBy: A Rameshkumar; individualCount: 1; sex: female; lifeStage: Adult; **Location:** continent: Asia; country: India; countryCode: IND; stateProvince: Tamil Nadu; municipality: Salem; locality: Yercaud; **Identification:** identifiedBy: A Rameshkumar; **Event:** samplingProtocol: Yellow pan trap; eventTime: 2014-08-06; habitat: Weedy field; **Record Level:** institutionID: ICAR-National Bureau of Agricultural Insect Resources; institutionCode: ICAR-NBAIR

#### Distribution

**Figure 16.**
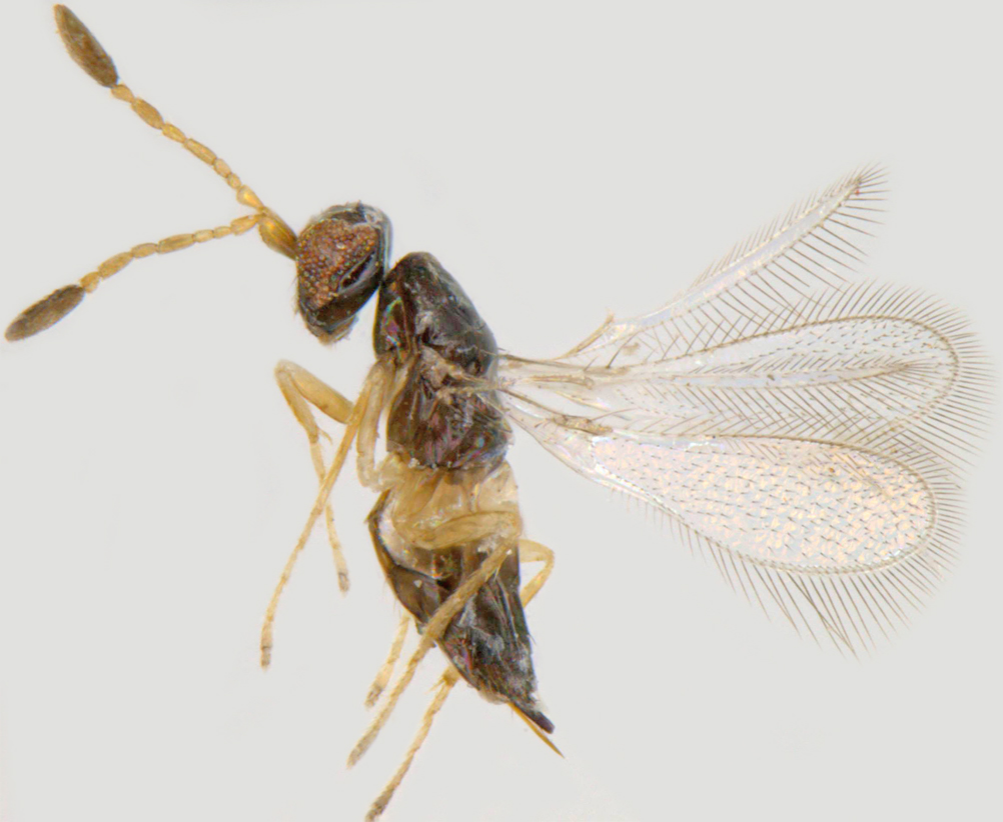
*Schizophragma* sp., lateral view.

*Schizophragma* sp. (Fig. 16​) was recorded from Meghalaya (Ramesh Kumar et al. 2013). We record it here from Tamil Nadu.

### Stethynium sp.

#### Materials

- **Type status:** Other material. **Occurrence:** recordedBy: A Rameshkumar; individualCount: 3; sex: female; lifeStage: Adult; **Location:** continent: Asia; country: India; countryCode: IND; stateProvince: Kerala; municipality: Calicut; locality: East hills; **Identification:** identifiedBy: A Rameshkumar; **Event:** samplingProtocol: Yellow pan trap; eventDate: 2014-04-25; habitat: Weedy field; **Record Level:** institutionID: ICAR-National Bureau of Agricultural Insect Resources; institutionCode: ICAR-NBAIR

#### Distribution

**Figure 17.**
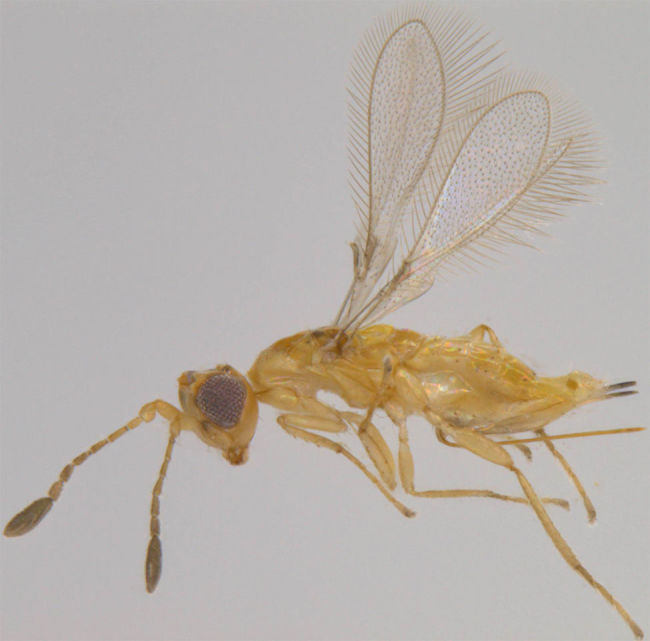
*Stethynium* sp., lateral view.

*Stethynium* sp. (Fig. 17​​) was recorded from Delhi, Gujarat, Karnataka and Uttar Pradesh (Subba Rao and Hayat 1983). We record it here from Kerala.

## Supplementary Material

XML Treatment for Acmopolynema indochinense

XML Treatment for Acmopolynema malabaricum

XML Treatment for Acmopolynema problema

XML Treatment for Alaptus sp.

XML Treatment for Camptoptera matcheta

XML Treatment for Dicopus longipes

XML Treatment for Dicopus noyesi

XML Treatment for Eofoersteria sp.

XML Treatment for Eubroncus sp.

XML Treatment for Gonatocerus monticolus

XML Treatment for Gonatocerus trialbifuniculatus

XML Treatment for Kikiki huna

XML Treatment for Litus sutil

XML Treatment for Litus triapitsyni

XML Treatment for Ooctonus nigrotestaceus

XML Treatment for Schizophragma sp.

XML Treatment for Stethynium sp.

## Figures and Tables

**Figure 12a.**
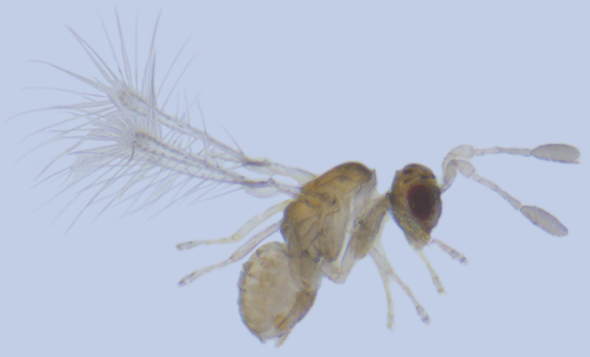
Female, lateral view

**Figure 12b.**
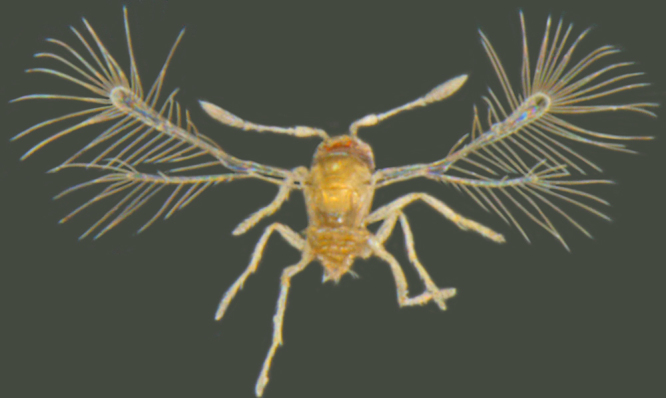
Female, dorsal view

## References

[B1177396] Brown B. V. (1993). A further chemical alternative to critical point drying for preparing small (or large) flies. Fly Times.

[B1188602] Hayat M., Anis S. B. (1999). The Indian species of *Acmopolynema* with notes on *Acanthomymar* (Hymenoptera: Chalcidoidea: Mymaridae). Oriental Insects.

[B1188612] Hayat M., Khan F. R. (2009). First record of *Eubroncus* from India (Hymenoptera: Chalcidoidea: Mymaridae) with description of a new species. Journal of Threatened Taxa.

[B1259796] Huber J. T. (2009). The genus *Dicopomorpha* (Hymenoptera, Mymaridae) in Africa and a key to *Alaptus*-group genera. Zookeys.

[B1183573] Huber J. T., Noyes J. S. (2013). A new genus and species of fairyfly, *Tinkerbella* nana (Hymenoptera, Mymaridae), with comments on its sister genus *Kikiki*, and discussion on small size limits in arthropods. Journal of Hymenoptera Research.

[B1183584] Huber J. T, Mendel Z, Protasov A, LaSalle J (2006). Two new Australian species of *Stethynium* (Hymenoptera: Mymaridae), larval parasitoids of *Ophelimus
maskelli* (Ashmead) (Hymenoptera: Eulophidae) on *Eucalyptus*. Journal of Natural History.

[B1188622] Manickavasagam S., Palanivel S. (2013). First report of two mymarid genera, *Cleruchus* Enock and *Kikiki* Huber and Beardsley (Hymenoptera: Mymaridae) from India. Journal of Biological Control.

[B1183594] Manickavasagam S, Rameshkumar A (2011). First report of three genera of fairyflies (Hymenoptera: Mymaridae) from India with description of a new species of *Dicopus* and some other records. Zootaxa.

[B1183604] Manickavasagam S, Rameshkumar A (2013). A Checklist of Mymaridae (Hymenoptera: Chalcidoidea) of India. Madras Agricultural Journal.

[B1183614] Manickavasagam S, Rameshkumar A, Rajmohana K (2011). First report of four species of fairyflies from India, key to Indian species of four genera and additional distributional records of Mymaridae (Hymenoptera: Chalcidoidea). Madras Agricultural Journal.

[B1183624] Noyes J. S (1982). Collecting and preserving chalcid wasps (Hymenoptera: Chalcidoidea).. Journal of Natural History.

[B1259786] Pricop E., Andriescu I (2011). *Dicopus
minutissimus* Enock (Hymenoptera: Mymaridae), representative of a genus and species new to Romania, with notes on other species.. North-Western Journal of Zoology.

[B1183652] Rameshkumar A, Manickavasagam S, Jebanesan A (2011). Diversity and new distributional records of fairyflies (Hymenoptera: Chalcidoidea: Mymaridae) from the state of Kerala, India. Plant Archives.

[B1183643] Ramesh Kumar A, Manickavasagam S, Poorani J, Malathi C (2013). Indian Genera of Mymaridae. http://www.nbair.res.in/IndianMymaridae/index.php.

[B1183662] Rehmat T, Anis S. B, Hayat M (2009). Record of the genus *Litus* Haliday (Hymenoptera: Chalcidoidea: Mymaridae) from India, with description of two new species. Journal of Threatened Taxa.

[B1183672] Subba Rao B. R (1984). Description of new species of Oriental Mymaridae and Aphelinidae (Hymenoptera: Chalcidoidea). Proceedings of the Indian Academy of Sciences (Animal Sciences).

[B1183682] Subba Rao B. R (1989). On a collection of Indian Mymaridae (Chalcidoidae: Hymenoptera). Hexapoda.

[B1183692] Subba Rao B. R, Hayat M (1983). Key to the genera of Oriental Mymaridae, with a preliminary catalog (Hymenoptera: Chalcidoidea). Contributions of the American Entomological Institute.

[B1259806] Triapitsyn S. V. (2013). On the occurrence of *Kikiki
huna* Huber (Hymenoptera: Mymaridae) in Argentina. Acta zoológica lilloana.

[B1183702] Triapitsyn S. V, Berezovskiy V. V (2007). Review of the Oriental and Australian species of *Acmopolynema*, with taxonomic notes on *Palaeoneura* and *Xenopolynema* stat. rev. and description of a new genus (Hymenoptera: Mymaridae). Zootaxa.

[B1183712] Zeya S. B, Hayat M (1995). A revision of the Indian species of *Gonatocerus* Nees (Hymenoptera: Mymaridae). Oriental Insects.

